# Entering a New Era of Body Indices: The Feasibility of a Body Shape Index and Body Roundness Index to Identify Cardiovascular Health Status

**DOI:** 10.1371/journal.pone.0107212

**Published:** 2014-09-17

**Authors:** Martijn F. H. Maessen, Thijs M. H. Eijsvogels, Rebecca J. H. M. Verheggen, Maria T. E. Hopman, André L. M. Verbeek, Femmie de Vegt

**Affiliations:** 1 Department of Physiology, Radboud University Medical Center, Nijmegen, The Netherlands; 2 Department for Health Evidence, Radboud University Medical Center, Nijmegen, The Netherlands; 3 Department of Cardiology, Hartford Hospital, Hartford, Connecticut, United States of America; University of Florida, United States of America

## Abstract

**Background:**

The Body Mass Index (BMI) and Waist Circumference (WC) are well-used anthropometric predictors for cardiovascular diseases (CVD), but their validity is regularly questioned. Recently, A Body Shape Index (ABSI) and Body Roundness Index (BRI) were introduced as alternative anthropometric indices that may better reflect health status.

**Objective:**

This study assessed the capacity of ABSI and BRI in identifying cardiovascular diseases and cardiovascular disease risk factors and determined whether they are superior to BMI and WC.

**Design and Methods:**

4627 Participants (54±12 years) of the Nijmegen Exercise Study completed an online questionnaire concerning CVD health status (defined as history of CVD or CVD risk factors) and anthropometric characteristics. Quintiles of ABSI, BRI, BMI, and WC were used regarding CVD prevalence. Odds ratios (OR), adjusted for age, sex, and smoking, were calculated per anthropometric index.

**Results:**

1332 participants (27.7%) reported presence of CVD or CVD risk factors. The prevalence of CVD increased across quintiles for BMI, ABSI, BRI, and WC. Comparing the lowest with the highest quintile, adjusted OR (95% CI) for CVD were significantly different for BRI 3.2 (1.4–7.2), BMI 2.4 (1.9–3.1), and WC 3.0 (1.6–5.6). The adjusted OR (95% CI) for CVD risk factors was for BRI 2.5 (2.0–3.3), BMI 3.3 (1.6–6.8), and WC 2.0 (1.6–2.5). No association was observed for ABSI in both groups.

**Conclusions:**

BRI, BMI, and WC are able to determine CVD presence, while ABSI is not capable. Nevertheless, the capacity of BRI as a novel body index to identify CVD was not superior compared to established anthropometric indices like BMI and WC.

## Introduction

Overweight and obesity are the fifth leading cause of global death and are an increasing worldwide health problem. In 2008, more than 1.4 billion adults suffered from overweight and within this group approximately 36% adults is obese [1]. Overweight and obesity are associated with an increased risk for cardiovascular diseases (CVD), type 2 diabetes mellitus and premature death [2]–[9]. Therefore, an early detection of overweight or obesity is considered necessary to prevent CVD [10].

The Body Mass Index (BMI) and Waist Circumference (WC) are currently recommended by several guidelines to classify overweight and obesity [10], [11]. Indeed, an increase in BMI or WC has been shown to be a risk factor for CVD [10]. However, previous studies also demonstrated that the discriminative capacity of BMI is not optimal, as this calculation cannot distinguish between adipose tissue and lean body mass [12]–[16]. WC alternatively, has been shown to be a good predictor for abdominal adipose tissue [17], [18], but currently it is unclear to what extent the range of WC depends on body size [19], [20]. This has led to the idea that by combining traditional measures (*e.g.* height, weight, BMI, or WC) a better body index could be designed, which takes body shape into account [21]–[24].

Recently, two new body indices have been introduced [22], [23]. In 2012, *Krakauer et al*. developed ‘A Body Shape Index’ (ABSI), which is based on waist circumference (*m*), BMI (*kg*⋅*m^−2^*), and height (*m*) [22]. According to the authors, a high ABSI relates to a greater fraction of abdominal adipose tissue and appears to be a significant risk factor for premature death [22]. Other studies have suggested that ABSI is able to predict the onset of diabetes mellitus [25] and that it could be used to evaluate physical health status of adolescents [26]. In 2013, *Thomas et al*. developed the Body Roundness Index (BRI), which is a new geometrical index that combines height (*m*) and waist circumference (*m*) to predict the percentage of body fat and to evaluate health status [23]. However, it is unknown whether ABSI and BRI can determine the presence or risk of cardiovascular diseases.

Therefore, the aim of this study was to assess the capacity of the novel indices A Body Shape Index (ABSI) and Body Roundness Index (BRI) to identify cardiovascular diseases and cardiovascular disease risk factors in the Dutch population and to determine whether these anthropometric indices are superior to the Body Mass Index and Waist Circumference. We postulate that ABSI and BRI can better identify CVD than BMI or WC.

## Methods

### Ethics Statement

The study adhered to the Declaration of Helsinki. The Local Committee on Research Involving Human Subjects (CMO) of the region Arnhem and Nijmegen approved the study, and all subjects gave their written informed consent.

### Study design and study population

The Nijmegen Exercise Study (www.nijmegenexercisestudy.com) is a large-population based study conducted by the Department for Health Evidence and the Department of Physiology of the Radboud University Medical Center in June 2011. The overall aim of the Nijmegen Exercise Study is to examine the effects of physical activity on general health and various disease outcomes in the general population.

In 2011, participants of the International Nijmegen Four Days Marches, the largest multi-day walking event in the world, were eligible to participate in the Nijmegen Exercise Study. By means of a passive recruitment strategy (Four Days Marches newsletter and internet advertisement), Dutch speaking adults were invited to complete an online survey. Participants were asked about their date of birth, anthropometric measures (weight, height, and WC), and whether they were diagnosed with CVD. In addition, participants were questioned about their lifestyle factors (physical activity and smoking habits).

### Anthropometric measures

#### Self-reported body height, weight, and waist circumference

Participants reported their height (centimetre), waist circumference (centimetre), and body weight (kilogram) in the online survey. Each anthropometric measurement was accompanied with detailed instructions. These data were used to calculate the BMI, ABSI, and BRI according to standardized formulas. BMI was based on weight (*kg*) and height (*m*) and calculated using formula [1] [27].
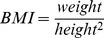
(1)


#### A body shape index

The ABSI was based on height (*m*), BMI (*kg*⋅*m^−2^*), and waist circumference (*m*) and calculated using formula [2] [22].
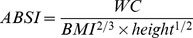
(2)


#### The Body Roundness Index

The BRI was based on height (*m*) and waist circumference (*m*). First, the eccentricity (*ε*) of the body was determined using formula [3]. Eccentricity (non-dimensional value) quantifies the degree of circularity of an ellipse, and it ranges between zero (perfect circle) to one (a vertical line) [23].
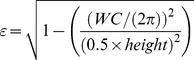
(3)


Subsequently, the BRI was calculated using formula [4]. Described by *Thomas et al*., values closer to 1 are related to leaner individuals, whereas larger values are associated with rounder individuals [23].
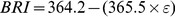
(4)


### Presence of cardiovascular diseases

Participants were asked whether their physician diagnosed (yes/no) presence of CVD (1. myocardial infarction and 2. stroke) or CVD risk factors (1. hypertension and 2. hypercholesterolemia). Participants were categorized in the CVD or CVD risk factor group if one of these questions were replied positively, while they were allocated to the control group if all answers were negative. Participants who reported CVD as well as CVD risk factors were allocated to the CVD group.

### Lifestyle factors

All participants reported whether they had followed a training program prior to the Nijmegen Four Days Marches and/or whether they performed other sport activities (*e.g.* playing soccer, tennis, etc.). Based on the duration (hours/week), frequency, and intensity (low, moderate, high) of these exercise activities, the average time and intensity level of physical activity per day was calculated. The recommendations of the American Heart Association were used to determine if a participant fulfilled the physical activity criteria [28].

Participants reported whether they were current smoker, ex-smoker or non-smokers and were categorized as (ex)-smoker or non-smoker.

### Calculations and statistical analysis

Group characteristics were analysed using descriptive techniques. Continuous variables (age, height, weight, ABSI, BMI, BRI, and WC) were reported in tables as mean and differences between the CVD, CVD risk factor, and control group were analysed using one-way analysis of variance (ANOVA) with a Bonferroni post-hoc test. Categorical data were reported in proportions and differences tested by *Pearson’s* chi-squared test (sex, physical activity, myocardial infarction, stroke, hypertension, hypercholesterolemia, and smoking). Statistical significance was assumed at *P*<0.05.

Pairwise correlation coefficients between the continuous variables height, weight, ABSI, BMI, BRI, and WC were assessed by calculating Pearson correlation coefficients. To examine the discriminative power of the anthropometric indices for CVD or CVD risk factors the Area under the Receiver Operating Characteristic (AROC) curves were calculated. The AROC represents a measure of accuracy of an anthropometric index to discriminate between subjects with or without CVD or CVD risk factors [29].

Quintiles of BMI and BRI were created and the prevalence of CVD and CVD risk factors was calculated in each quintile. Since ABSI is strongly correlated with age and sex [22], ABSI was stratified for age (per year), after which ABSI quintiles were determined within each age group for males and females separately. To calculate the prevalence of CVD and CVD risk factors per ABSI quintile, subjects within the same ABSI quintile were merged. For WC the quintiles were stratified by sex and the prevalence of CVD and CVD risk factors in each quintile was calculated. Logistic regression was used to estimate the odds ratio for suffering from CVD or CVD risk factors per quintile, both unadjusted and adjusted for sex, age, and lifestyle factors (smoking and physical activity). For all four anthropometric indices, the lowest quintile was set as reference. All statistical analyses were performed using SPSS 20.0 (IBM Corp. Released 2011. IBM SPSS Statistics for Windows, Version 20.0. Armonk, NY: IBM Corp) software.

## Results

**Figure 1:**
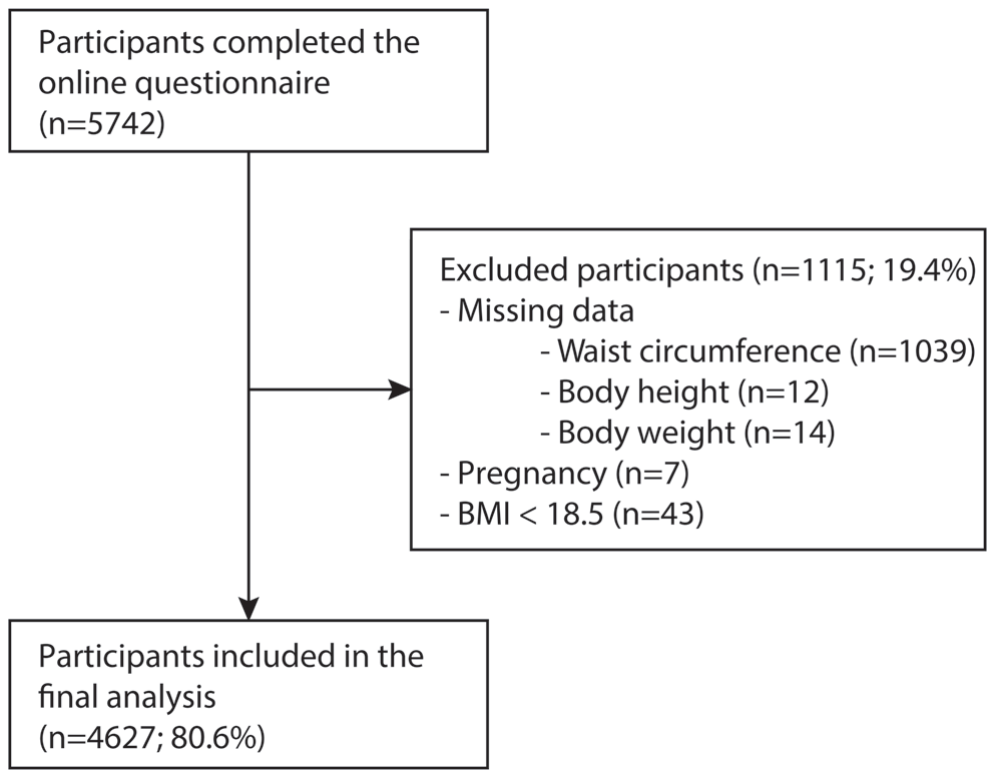
Flowchart enrolment of the study population. 5742 participants completed the online questionnaire. 1115 participants were excluded because of missing data (n = 1065), pregnancy (n = 7), or BMI <18.5 (n = 43). The final group sample consisted of 4627 participants.

5742 participants completed the online questionnaire. After the exclusion of participants who were pregnant, had underweight (calculated BMI of <18.5), or with missing data on anthropometric measures, the study population consisted of 4627 (80.6%) participants (Figure 1). This included 2425 men (57.5±11.4 years of age) and 2202 women (50.2±12.6 years of age). 1332 (27.7%) participants reported CVD or CVD risk factors, of which 32.9% (438) were female. Hypertension and hypercholesterolemia were reported in respectively 19.5% and 14.6% of the study population. Myocardial infarction and stroke were reported in respectively 2.6% and 1.4% of the study population (Table 1).

**Table 1:** Characteristics of the total study population and according to cardiovascular disease status.

Parameters	Group			
*n*	*4627*			
Sex, % females (*n*)	47.6 (2202)			
CVD and risk factors, %yes (*n*)	23.2 (1332)			
MI, %yes (*n*)	2.6 (121)			
Stroke, %yes (*n*)	1.4 (65)			
Hypertension, %yes (*n*)	19.5 (903)			
Hypercholesterolemia, %yes (*n*)	14.6 (674)			
	**CVD**	**CVD risk factors**	**Controls**	**P-value**
*n*	*179*	*1153*	*3295*	*–*
Sex, % females (*n*)	9.5 (17)* ^,^ **	36.5 (421)*	53.5 (1764)	<0.001
Age (years)	62.9 (±8.3)* ^,^ **	59.6 (±8.8)*	51.6 (±13.0)	<0.001
Height (cm)	176.4 (±7.3)* ^,^ **	174.4 (±8.8)	174.5 (±9.1)	0.020
ABSI (m^11/6^⋅kg^−2/3^)	0.084 (±0.005)* ^,^ **	0.082 (±0.005)*	0.081 (±0.006)	<0.001
BMI (kg⋅m^−2^)	26.2 (±3.0)* ^,^ **	25.6 (±3.1)*	24.4 (±3.0)	<0.001
BRI	4.5 (±1.1)* ^,^ **	4.2 (±1.1)*	3.7 (±1.2)	<0.001
Weight (kg)	81.6 (±11.5)* ^,^ **	78.1 (±13.1)*	74.5 (±12.6)	<0.001
WC (cm)	97.9 (±9.0)* ^,^ **	94.4 (±10.3)*	89.8 (±11.0)	<0.001
Norm PA (%yes [*n*])	66.5 (119)	67.4 (777)*	59.2 (1949)	<0.001
Smoking (%yes [n])	71.5 (128)* ^,^ **	61.6 (710)*	49.5 (1631)	<0.001

Abbreviations: ABSI, A Body Shape Index; BRI, Body Roundness Index; BMI, Body Mass Index; MI, Myocardial Infarction; norm PA, norm physical activity;

Data presented as mean (SD) or proportion (number).

*significantly different from ‘*Controls’*;

** significantly different from ‘ *CVD risk factors*’.

### CVD, CVD risk factor and control group characteristics

The participants with CVD and CVD risk factors were older (62.9±8.3 *vs.* 59.6±8.8 *vs.* 51.6±13.0 year; *P*<0.001) compared the control group. The CVD and CVD risk factor group reported a higher body weight (81.6 (±11.5) *vs.* 78.1 (±13.1) *vs.* 74.5±12.6 kg; *P*<0.001) and smoked more often (71.5% *vs.* 61.6% *vs.* 49.5%; *P*<0.001). There were more participants physically active within the CVD risk factor group compared the control group (67.4 *vs.* 59.2; *P*<0.001), whereas the CVD and control group were comparable (66.5 *vs.* 59.2; *P* = 0.06) (Table 1). Furthermore, participants with CVD and CVD risk factors demonstrated higher ABSI, BMI, BRI, and WC values compared to the control group (Table 1).

### Correlation and AROC scores

**Figure 2:**
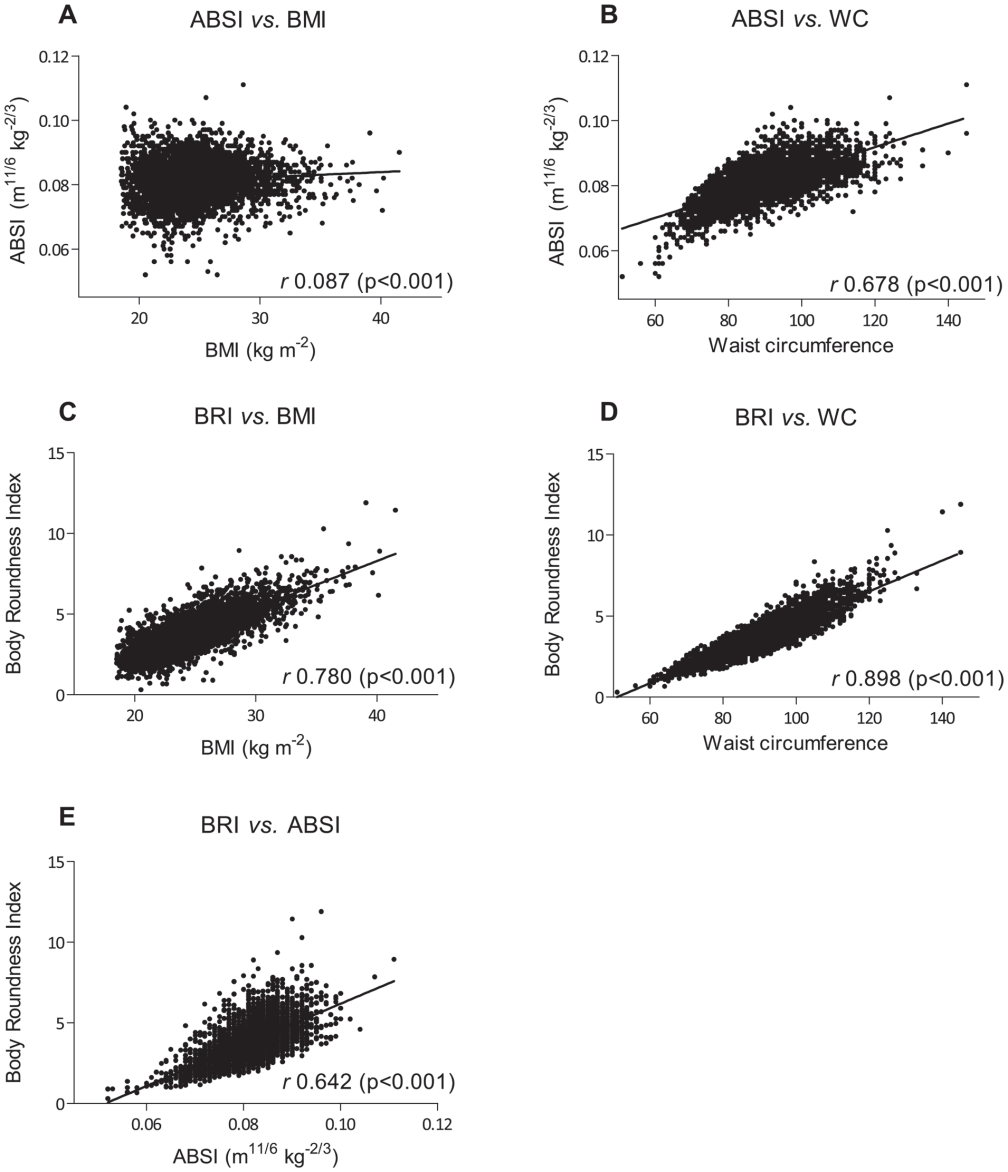
Correlations between ABSI, BMI, BRI, and WC. **A.** Correlation between ABSI and BMI. Increase in ABSI and increase in BMI show a poor yet significant correlation (*r* = 0.087, *P*<0.001). **B.** Correlation between ABSI and WC. Increase in ABSI and increase in WC show a significant correlation (*r* = 0.678, *P*<0.001). **C.** Correlation between BRI and BMI. Increase in BRI and increase in BMI show a significant correlation (*r* = 0.780, *P*<0.001). **D.** Correlation between BRI and WC. Increase in BRI and increase in WC show a significant correlation (*r = *0.898, *P*<0.001). **E.** Correlation between BRI and ABSI. Increase in BRI and increase in ABSI show a significant correlation (*r = *0.642, *P*<0.001).

**Figure 3:**
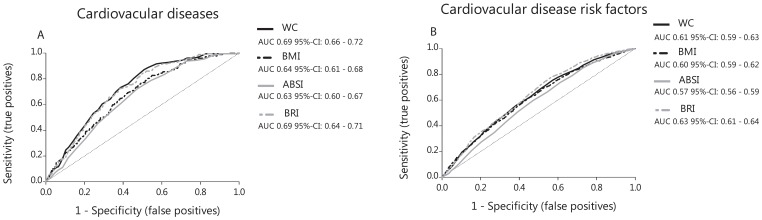
The discriminatory power of ABSI, BRI, BMI, and WC between subjects with or without CVD or CVD risk factors. **A.** Area under the Receiver Operating Characteristic curve of ABSI, BRI, BMI, and WC to identify subjects with cardiovascular diseases. **B.** Area under the Receiver Operating Characteristic curve of ABSI, BRI, BMI, and WC to identify subjects with cardiovascular disease risk factors.

ABSI and BRI were positively and significantly correlated to height, weight, BMI and WC (Table 2; Figure 2). Within the CVD group, the AROC scores were 0.63 for ABSI, 0.68 for BRI, 0.64 for BMI, and 0.69 for WC (Figure 3A). Within the CVD risk factor group, the AROC scores were 0.57 for ABSI, 0.63 for BRI, 0.60 for BMI, and 0.61 for WC (Figure 3B).

**Table 2:** Correlations between body size and shape.

	Height	Weight	WC	ABSI	BRI	BMI
Height	**1**					
Weight	0.69**	**1**				
WC	0.40**	0.79**	**1**			
ABSI	0.18**	0.18**	0.68**	**1**		
BRI	−0.04*	0.54**	0.90**	0.64**	**1**	
BMI	0.11**	0.79**	0.75**	0.09**	0.77**	**1**

ABSI: A Body Shape Index; BRI: Body Roundness Index; BMI: Body Mass Index; WC; Waist Circumference.

Correlation coefficients between height, weight, ABSI, BMI, BRI, and WC among the NES study population (n = 4627). **Correlation is significant at the 0.01 level (2-tailed). *Correlation is significant at the 0.05 level (2-tailed).

### Prevalence of CVD and CVD risk factors

The prevalence of CVD increased per quintile for all four anthropometric indices (1^st^ quintile *vs*. 5^th^ quintile): ABSI 3.9% *vs*. 4.3%, BRI 0.8% *vs*. 6.5%, BMI 1.0% *vs*. 6.1%, WC 1.0% *vs*. 6.1% (*P*<0.05) (Table 3A). The prevalence of CVD risk factors increased per quintile for all four anthropometric indices (1^st^ quintile *vs*. 5^th^ quintile): ABSI 23.2% *vs*. 24.1%, BRI 11.6% *vs*. 36.9%, BMI 15.0% *vs*. 34.2%, WC 15.0% *vs*. 34.2% (*P*<0.05) (Table 3B).

**Table 3:** Prevalence of CVD and CVD risk factors in quintiles of ABSI, BRI, BMI, and WC.

*Cardiovascular diseases*				
Quintile	ABSI	BRI	BMI	WC
1 (%CVD [n])	3.9 (34)	0.8 (7)	1.0 (9)	1.0 (9)
2 (%CVD [n])	3.4 (31)	1.9 (18)	2.4 (22)	2.4 (22)
3 (%CVD [n])	3.8 (35)	3.8 (35)	4.4 (41)	4.4 (41)
4 (%CVD [n])	4.0 (37)	6.4 (59)	5.4 (50)	5.4 (50)
5 (%CVD [n])	4.3 (42)	6.5 (60)	6.1 (57)	6.1 (57)
***Cardiovascular disease risk factors***				
**Quintile**	**ABSI**	**BRI**	**BMI**	**WC**
1 (%CVD [n])	23.2 (204)	11.6 (107)	15.0 (139)	15.0 (139)
2 (%CVD [n])	25.9 (239)	20.1 (186)	21.4 (198)	21.4 (198)
3 (%CVD [n])	25.8 (239)	29.7 (275)	25.6 (237)	25.6 (237)
4 (%CVD [n])	25.5 (235)	26.4 (244)	28.4 (262)	28.4 (262)
5 (%CVD [n])	24.1 (236)	36.9 (341)	34.2 (317)	34.2 (317)

ABSI: A Body Shape Index; BRI: Body Roundness Index; BMI: Body Mass Index; WC; Waist Circumference.

Data presented as mean (SD) or proportion (number).

### Odds of cardiovascular diseases

The unadjusted odds of CVD prevalence increased with increasing quintiles for ABSI (OR_unadjusted = _1.1 [0.7−1.8]), BRI (OR_unadjusted = _9.1 [4.1−20.1]), BMI (OR_unadjusted = _6.7 [3.3−13.5]), and WC (OR_unadjusted = _3.4 [1.9−6.3]) (Table 4). Physical activity was non-significant in the logistic regression analysis and was therefore excluded from further analysis. After adjustment for age, sex, and smoking, the odds ratio of CVD was still significant for BRI (OR_adjusted_ 3.2 [1.4−7.2]), BMI (OR_adjusted = _3.3 [1.6−6.8]), and WC (OR_adjusted = _3.0 [1.6−5.6]). However, the odds ratios did not vary across the ABSI quintiles (OR_adjusted = _1.1 [0.7−1.8]) (Table 4).

**Table 4:** The unadjusted odds of CVD and CVD risk factor prevalence for ABSI, BRI, BMI, and WC.

*Cardiovascular diseases*				
Quintile	ABSI	BRI	BMI	WC
1 (reference)	1	1	1	1
2	0.9 (0.5−1.4)	2.6 (1.1−6.3)*	2.5 (1.1−5.4)*	2.6 (1.4−4.9)^*Φ<*^
3	1.0 (0.6−1.6)	5.2 (2.3−11.7)^*Φ<*^	4.7 (2.3−9.8)^*Φ<*^	2.7 (1.5−5.0)^*Φ<*^
4	1.0 (0.6−1.7)	8.9 (4.1−19.7)^*Φ<*^	5.8 (2.9−12.0)^*Φ<*^	3.4 (1.9−6.3)^*Φ<*^
5	1.1 (0.7−1.8)	9.1 (4.1−20.1)^*Φ<*^	6.7 (3.3−13.5)^*Φ<*^	3.4 (1.9−6.3)^*Φ<*^
***Cardiovascular disease risk factors***				
**Quintile**	**ABSI**	**BRI**	**BMI**	**WC**
1 (reference)	1	1	1	1
2	1.2 (0.9−1.4)	1.9 (1.5−2.5)^*Φ*^	1.5 (1.2−2.0)^*Φ<*^	1.4 (1.1−1.8)^*Φ<*^
3	1.1 (0.9−1.4)	3.2 (2.5−4.1)^*Φ<*^	1.9 (1.5−2.5)^*Φ<*^	1.7 (1.3−2.1)^*Φ<*^
4	1.1 (0.9−1.4)	2.7 (2.1−3.5)^*Φ<*^	2.2 (1.8−2.8)^*Φ<*^	1.8 (1.5−2.3)^*Φ<*^
5	1.1 (0.8−1.3)	4.5 (3.5−5.7)^*Φ<*^	2.9 (2.3−3.7)^*Φ<*^	2.4 (2.0−3.0)^*Φ<*^

ABSI: A Body Shape Index; BRI: Body Roundness Index; BMI: Body Mass Index; WC; Waist Circumference.

Ranges in parentheses are 95% confidence intervals. The between cut points are 0.077, 0.080, 0.083, and 0.086 for ABSI; 2.8, 3.5, 3.9, 4.7 for BRI; 22.1, 23.7, 25.2, and 27.1 for BMI; 0.89, 0.94, 0.98, 1.04 for WC (males); 0.78, 0.82, 0.87, 0.94 for WC (females). *Significant at P<0.05; ^*Φ*^Significant at P<0.01.

### Odds of cardiovascular disease risk factors

The unadjusted odds of CVD risk factor prevalence increased with increasing quintiles for ABSI (OR_unadjusted = _1.1 [0.8−1.3]), BRI (OR_unadjusted = _4.5 [3.5−5.7]), BMI (OR_unadjusted = _2.9 [2.3−3.7]), and WC (OR_unadjusted = _2.4 [2.0−3.0]) (Table 5). After adjustment for age, sex, and smoking, the odds ratio of CVD was still significant for BRI (OR_adjusted_ 2.5 [2.0−3.3]), BMI (OR_adjusted = _2.4 [1.9−3.1]), and WC (OR_adjusted = _2.0 [1.6−2.5). However, the odds ratios did not vary across the ABSI quintiles (OR_adjusted = _1.1 [0.9−1.3]) (Table 5).

**Table 5:** The odds of CVD and CVD risk factor prevalence for quintiles of ABSI, BRI, BMI, and WC adjusted for sex, age, and smoking.

*Cardiovascular diseases*				
Quintile	ABSI	BRI	BMI	WC
1 (reference)	1	1	1	1
2	0.8 (0.5−1.4)	1.3 (0.5−3.2)	1.5 (0.7−3.4)	2.5 (1.3−4.8)^*Φ<*^
3	1.0 (0.6−1.6)	2.1 (0.9−4.8)	2.5 (1.2−5.2)*	2.3 (1.2−4.4)^*Φ<*^
***Cardiovascular disease risk factors***				
**Quintile**	**ABSI**	**BRI**	**BMI**	**WC**
1 (reference)	1	1	1	1
2	1.2 (0.9−1.5)	1.3 (1.0−1.7)	1.3 (1.0−1.7)*	1.3 (1.0−1.6)*
3	1.2 (0.9−1.5)	2.0 (1.6−2.6)^*Φ<*^		
4	1.2 (0.9−1.4)	1.6 (1.2−2.1)^*Φ<*^	1.7 (1.4−2.2)^*Φ<*^	1.5 (1.2−1.9)^*Φ<*^
5	1.1 (0.9−1.3)	2.5 (2.0−3.3)^*Φ<*^	2.4 (1.9−3.1)^*Φ<*^	2.0 (1.6−2.5)^*Φ<*^

ABSI: A Body Shape Index; BRI: Body Roundness Index; BMI: Body Mass Index; WC; Waist Circumference.

Ranges in parentheses are 95% confidence intervals. The between cut points are 0.077, 0.080, 0.083, and 0.086 for ABSI; 2.8, 3.5, 3.9, 4.7 for BRI; 22.1, 23.7, 25.2, and 27.1 for BMI; 0.89, 0.94, 0.98, 1.04 for WC (males); 0.78, 0.82, 0.87, 0.94 for WC (females). *Significant at P<0.05; ^*Φ*^Significant at P<0.01.

## Discussion

We demonstrated that the prevalence of CVD and CVD risk factors significantly increased across the quintiles for ABSI, BRI, BMI, and WC. However, after adjusting for age, sex, and smoking only BRI, BMI, and WC could identify CVD or CVD risk factors and not ABSI. In contrast to our hypothesis, neither ABSI nor BRI are superior measures compared to BMI and WC to determine the presence of CVD or CVD risk factors. Our findings indicate that the novel index ABSI is not suitable for identifying CVD or CVD risk factors, while the BRI showed similar capabilities as BMI and WC.

### A Body Shape Index

The ABSI was created to produce a quantitative measure to estimate the health of body shape independently of body size (height, weight, and BMI) [22]. While *Krakauer et al*. demonstrated that ABSI predicts premature mortality better than BMI or WC [22], our results suggest that ABSI cannot be used to distinguish between individuals with and without CVD or CVD risk factors. Compared with BRI, BMI, and WC, the ABSI demonstrated the lowest predictive power and had no significant association with CVD or CVD risk factors prevalence after adjustment for age, sex, and smoking. Thus, ABSI is not a suitable measurement to identify CVD and CVD risk factors.

A possible explanation for the contrasting findings between our data and *Krakauer et al.,* is the endpoint variable, namely the prevalence of CVD and CVD risk factors *versus* premature death. Alternatively, subject characteristics may explain the dissimilarities between our study and the one of *Krakauer et al.* [30]. Our study population has approximately the same ABSI values as *Krakauer i.e.* 0.081±0.0058 m^11/6^⋅kg^−2/3^ *vs.* 0.081±0.0053 m^11/6^⋅kg^−2/3^, but surprisingly our study population had a lower BMI (3^rd^ quintile: 23.7–25.2 *vs.* 25.6–28.4) and lower WC (3^rd^ quintile females: 82–87 *vs*. 88–97; males: 94–98 *vs*. 94–101). The explanation why ABSI is similar is that our study population comprising Dutch subjects were taller compared to the American study population of *Krakauer et al*. The estimated average body height is 1.70 meters in the study of *Krakauer et al.*, whereas our study population had an average body height of 1.75 meters. This suggests that body height might confound the capacity of ABSI to identify CVD in our study population. Future studies should further investigate the limits of ABSI and especially study the impact of body height on the calculation of ABSI.

### Body Roundness Index

We are the first to study the capacity of BRI to identify CVD and CVD risk factors. The BRI was developed to predict both body fat and the percentage visceral adipose tissue by using WC in relation to height, which allows estimation of the shape of the human body figure as an ellipse or oval [23]. We demonstrated that the BRI is capable to identify CVD and CVD risk factors (Table 3). This is in agreement with previous studies [21], [24] which also related WC to body height (*i.e.* waist-to-height ratio [WtH-ratio]). The spearman rank test revealed a perfect nonlinear relation between BRI and WtH-ratio (*r* = 1; *P* = 0.00). This indicates that both body indices are related by an one-to-one nonlinear transformation and strictly means that it does not matter what index (BRI or WtH-ratio) is used to determine CVD. However, as demonstrated by *Thomas et al.,* the advantage of the BRI over the WtH-ratio is that it also can be used to estimate the amount of body fat percentage and gives therefore a better impression of physical health status. Although the adjusted OR of the BRI was higher compared to BMI and WC, this did not reach statistical significance. Nevertheless, we recommend that future studies should study the sensitivity of BRI to determine cardiovascular health risks in a clinical setting. With the development of computerized software that is accessible via internet websites, Tablets or Smartphone applications, novel body measures with complex algorithms can easily be used by physicians [22], [23]. Therefore, the BRI has the potential to improve the detection, evaluation, and progression of CVD and CVD risk factors.

### Methodological considerations

Our study compared the ABSI and BRI mutually and against the BMI and WC in a large heterogeneous study population. In our study, participants self-reported their weight and WC using our written instructions, whilst in the study of *Krakauer et al.* trained personnel measured WC. Although, it is likely that participants made some measurement errors, prior studies demonstrated that self-report of body weight and waist circumference is a validated and feasible method [31], [32]. We therefore suppose that self-reporting has had a minor influence on our findings. Additionally, the outcome variable (CVD) was self-reported; participants were only allowed to report CVD or CVD risk factors if they were diagnosed by a physician. Possibly some participants had a history of CVD and did not report this. This could have led to an underestimation of our results. Therefore, the capacity of BRI, BMI, and WC to identify CVD or CVD risk factors could somewhat be higher. Finally, one might argue that the cross-sectional nature of our study is suboptimal to study the predictive capacity of anthropometric characteristics. However, in our study design we demonstrated that the BRI as well as BMI and WC were able to identify participants with CVD. It is a first step in validating BRI in relation to CVD and therefore we believe that the used method is appropriate. In future studies, the longitudinal relation between BRI and CVD incidence should be studied.

### Conclusion

In the current study, we demonstrated that CVD and CVD risk factors prevalence increased per quintile across a heterogeneous population for ABSI, BRI, BMI, and WC. Nonetheless, only BRI, BMI, WC, and not ABSI, could significantly determine the presence of CVD and CVD risk factor after correction for sex, age and smoking. The capacity of BRI to mathematically model the human body shape gives an adequate impression of the cardiovascular health status, but was not superior to BMI or WC.
